# Optimization of the Split-Spinach Aptamer for Monitoring Nanoparticle Assembly Involving Multiple Contiguous RNAs

**DOI:** 10.3390/nano9030378

**Published:** 2019-03-06

**Authors:** Jack M. O’Hara, Dylan Marashi, Sean Morton, Luc Jaeger, Wade W. Grabow

**Affiliations:** 1 Department of Chemistry and Biochemistry, Seattle Pacific University, Seattle, WA 918119-1997, USA; oharaj@spu.edu (J.M.O.); marashid@spu.edu (D.M.); mortons@spu.edu (S.M.); 2 Department of Chemistry and Biochemistry, Biomolecular Science and Engineering Program, University of California, Santa Barbara, CA 93106-9510, USA; jaeger@ucsb.edu

**Keywords:** RNA nanotechnology, RNA self-assembly, light-up aptamer, RNA nanoparticle

## Abstract

The fact that structural RNA motifs can direct RNAs to fold and self-assemble into predictable pre-defined structures is an attractive quality and driving force for RNA’s use in nanotechnology. RNA’s recognized diversity concerning cellular and synthetically selected functionalities, however, help explain why it continues to draw attention for new nano-applications. Herein, we report the modification of a bifurcated reporter system based on the previously documented Spinach aptamer/DFHBI fluorophore pair that affords the ability to confirm the assembly of contiguous RNA strands within the context of the previously reported multi-stranded RNA nanoring. Exploration of the sequence space associated with the base pairs flanking the aptamer core demonstrate that fluorescent feedback can be optimized to minimize the fluorescence associated with partially-assembled RNA nanorings. Finally, we demonstrate that the aptamer-integrated nanoring is capable of assembling directly from transcribed DNA in one pot.

## 1. Introduction

RNA nanotechnology leverages the formation of programmable base pairs and regular three-dimensional folding patterns of structural RNA moieties to construct materials with precise, predefined shapes [1,2,3,4,5]. As a building material, RNA offers several unique benefits such as biocompatibility, the ability to generate or add diverse biological functions, and the potential to generate and assemble nanoparticles directly from DNA transcripts [6,7,8]. As a consequence, RNA nanoparticles have been used in a variety of applications including the delivery of therapeutics, as stable scaffolds for the attachment of functional moieties, and as molecular signaling devices [9,10,11,12]. While much progress has been made in the manufacturing of rationally designed RNA structures, few tools exist to monitor their assembly and/or allow the subsequent tracking of wholly formed nanoparticles. As the design and utilization of nanostructures with increased complexity continues to progress, new methods and systems intended to monitor and verify the assembly of nanoparticles will be required to advance the field of RNA nanotechnology further.

A promising strategy that has been developed to visualize RNA in recent years involves the use of light-up RNA aptamer/fluorophore pairs [13,14,15,16,17]. Several RNA-based aptamer/fluorophore pairs have been developed to allow the monitoring of any RNA transcript [18,19,20,21]. Fluorescent-based, label-free RNA tracking methods are thought to offer distinct advantages over other investigative strategies because they can be integrated non-intrusively into virtually any RNA of interest in a variety of different contexts [22,23,24,25,26,27]. The advent RNA aptamer/fluorophore pairs with tunable wavelengths and the development of user-friendly toolkits continues to provide greater accessibility and inspire new applications [28,29,30]. Multiple RNA light-up aptamers have been bifurcated in order to enable the monitoring of more than one RNA transcript [18,31,32,33]. Split-aptamer systems rely on the fact that the functional aptamer forms only when both non-functional halves combine in the presence of a small molecular fluorophore. Given the dynamic nature of aptamer assembly, such fluorogenic systems have opened up new applications that include high-throughput assays, controlled reporting of assembly and processing, the development of logic gates and molecular computation, and more [23,33,34,35]. While split-aptamers offer the ability to monitor the assembly of two unique RNA strands (or three if formed on a scaffold-strand), most RNA nanoparticles are composed of several unique strands of RNA. Thus the ability to confirm the assembly of multiple RNAs is an important requirement where the assembly of more complex nanoparticles is concerned. With this understanding in mind we set out to design a modified bifurcated platform that provides the capability to monitor the assembly of a nanoparticle comprised of more than two RNA strands.

Given its unique structure and demonstrated ability to be functionalized with a variety of RNA-based functional groups, we chose to integrate the split-Spinach aptamer into the previously reported RNA nanoring [36,37]. The nanoring/split-aptamer reporter system represents a significant expansion of previous uses of the light-up aptamer/fluorophore pairs which rely on the direct interactions at the secondary structure level alone. The goal of our system was to be able to detect tertiary contacts formed by RNAs not directly coupled to the split-aptamer. In this regard, our design focused on finding an optimal thermodynamic balance between split-aptamer assembly and nanoparticle assembly where the formation of the functional aptamer depended more on the assembly of the whole nanoring so that maximum fluorescence occurred only in the presence of the whole nanoparticle. Herein, we report a split-Spinach aptamer system with the ability to monitor the assembly of six strands of RNA in a single nanoparticle. We demonstrate that the integrated light-up aptamer exhibits significant sensitivity for fully- assembled over partially-assembled nanoring nanoparticles. In doing so, we believe this to be the first system developed with the ability to detect adjacent, long-range tertiary interactions as opposed to base pairing directly mediated by the aptamer itself. Finally, we discuss the particular design constraints associated with our system in order to suggest general considerations that could be applied to the development of future multi-stranded reporter systems.

## 2. Materials and Methods

### 2.1. Design and Synthesis of Split-Spinach Aptamer and Fluorophore

**Figure 1:**
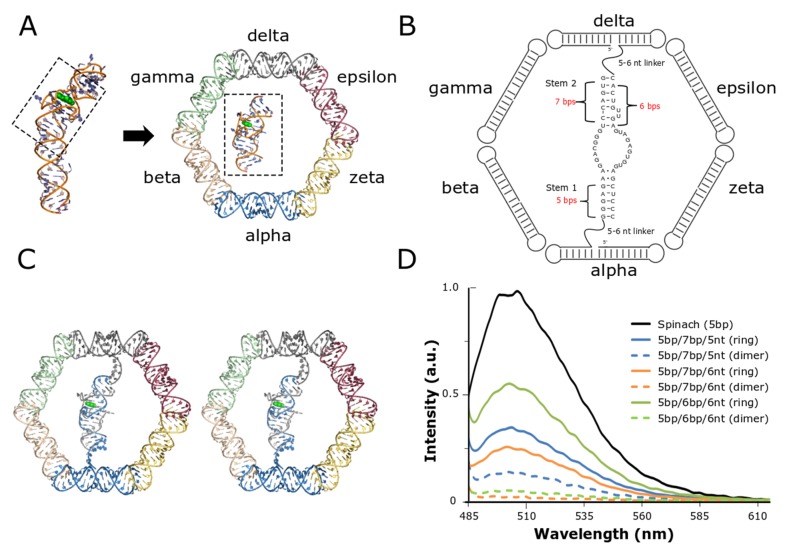
Design and integration of the split-Spinach aptamer and RNA nanoring. (**A**) The central portion of the Spinach aptamer crystallized by Huang et al. (PDB ID: 3IVK) [42] was placed in the interior of the previously reported RNA nanoring and grafted onto two of the nanoring’s opposing helical struts. Based on initial placement, the Spinach aptamer was modeled to contain two short stems and single-stranded linkers with variable lengths. (**B**) 2D diagram resulting from initial modification and modeling of the split-Spinach aptamer into the RNA nanoring. (**C**) Stereoview of split-aptamer integrated into RNA nanoring. (**D**) The variable stem and single-stranded linker lengths were tested via fluorescent spectroscopy in the presence of the light-up chromophore DFHBI. The combination containing 5- and 6-bp stems and 6-nt linkers (5bp/6p/6nt) showed the highest response and was therefore chosen as the initial base model for further refinement.

The previously published Spinach aptamer (PBD ID: 4TS2) [33,38] was modeled into the RNA nanoring [37] using the Swiss PDB-Viewer [39]. Placement of the aptamer inside the nanoring provided an initial estimate regarding stem and linker-strand lengths as well as optimal orientation of the aptamer. The split-Spinach strands were fused to two of the opposing nanoring struts (Figure 1A). Individual RNA strands were rationally-designed and evaluated for unintended secondary folding patterns prior to their synthesis and experimentation [40,41]. Sequences associated with helical regions of the nanoring struts were optimized to avoid secondary structures that would interfere with nanoring loops and/or the core of the Spinach aptamer—both sequence regions which could not be altered. DNA sequences, corresponding to the RNA sequences of interest, were designed by adding a T7 polymerase promoter site sequence (TTCTAATACGACTCACTATA) to the 5’ end of each RNA. Corresponding DNA templates and primers were purchased from Integrated DNA technologies (IDT, San Diego, CA, USA), amplified using *taq* DNA polymerase (Thermo Fisher Scientific, Waltham, MA, USA) via polymerase chain reaction (PCR), and isolated by a DNA purification kit (Epoch Life Sciences, Missouri, MO, USA). Transcription of amplified DNA was accomplished using T7 RNA polymerase (Thermo Fisher Scientific, Waltham, MA, USA) in vitro. The resulting transcripts were purified by polyacrylamide gel electrophoresis (PAGE) [8–10% polyacrylamide, 8 M urea, 1×(89 mM, pH 8.2) Tris Borate (TB)]. Excised gel fragments containing RNA were placed in Crush and Soak buffer (200 mM NaCl, 10 mM Tris pH 7.5, 1 mM filtrated Na_2_EDTA pH 8, water), shook overnight at 5 °C, and the RNA was isolated the next day via ethanol precipitation. The fluorophore, 3,5-difluoro-4-hydroxybenzylidene imidazolinone (DFHBI), was synthesized as previously reported according the protocol of the Paige research group [21]. A complete list of RNA sequences used in the study can be found in the Supporting Information.

### 2.2. Monitoring Nanoring Assembly

Assembly of the split-aptamer integrated nanoring was evaluated by native PAGE (40 mM HEPES, pH 8.2 buffer and 2 mM Mg(OAc)_2_) and fluorescent spectroscopy. RNAs were assembled by combining equimolar concentrations of RNA strands (at a concentration of 500 mM unless noted otherwise) and the snap cool process (2 min at 95 °C and 3 min on ice). After snap cooling, an association buffer was added to achieve a final concentration of 40 mM HEPES (pH 8.2), 1 mM Mg(OAc)_2_, and 50 mM KCl. This mixture was incubated at 37 °C for 20 min and evaluated by fluorescence spectroscopy with an LS 55 luminescence spectrometer (PerkinElmer, Waltham, MA, USA). DFHBI was added (either before or after incubation) to final concentration of 1 mM. Samples were loaded into a 15 uL quartz cuvette (Starna Cells, Inc., Atascadero, CA, USA) and excited at 469 nm. Emission was recorded at 509 nm. Assembly products were also analyzed by a gel shift assay. Products were loaded into a 7% polyacrylamide gel of 1× HEPES (40 mM HEPES) buffer and 1 mM Mg(OAc)_2_. Gels were run at 6 W for 3−4 h at 4 °C. Gels were stained with Sybr Gold (Invitrogen, Carlsbad, CA, USA) and imaged using a FluoroChemQ gel imager (Protein Simple, San Jose, CA, USA).

### 2.3. Co-Transcriptional Assembly

Amplified DNA (0.35 μM of each individual strand) for the RNA ring pieces and/or aptamer were added to 5× co-transcription buffer (DTT (100 mM), NTPs (25 mM each), IPP (0.1 u/μL), RNasin (40 u/μL), and T7 RNA polymerase (20-120U)). The amount of T7 RNA polymerase was normalized to the total amount of DNA in each reaction mixture. The total volume of reaction mixtures was 20 μL. In a typical experiment, reaction mixtures were incubated at 37 °C for 60 min. After incubation, 3 μL of DNase I (1 u/μL) was added to each reaction mixture and then incubated for an additional 15 min at 37 °C. Aliquots of each reaction mixture were evaluated by fluorescence (17 μL) and/or by gel electrophoresis (3 μL) as described above. All fluorescence signals were normalized to the 5 base pair (bp) full-length Spinach aptamer reported by Huang et al which was used as a control in each individual experiment conducted [42].

## 3. Results & Discussion

### 3.1. Initial Design of the Split-Spinach/Nanoring System

The overall structural architecture of the RNA nanoring provided a unique opportunity to place a functional split-aptamer in the interior of the ring (Figure 1). The ability to rationally design and integrate the split-Spinach/fluorophore system into the nanoring was made possible because of the previously reported crystal structure of the full-length Spinach aptamer (PBD ID: 4TS2)—which was essential for evaluating its potential placement within the interior of the six-membered RNA nanoring in silico (Figure 1A) [36,42]. The two main stems flanking, and responsible for stabilizing, the fluorophore binding pocket were both shortened to approximately the same length in order to allow the fully-formed aptamer to sit comfortably in the middle of the interior region of the nanoring (Figure 1A). Previous reports revealed that one of the closing stems responsible for stabilizing the aptamer’s core—formed between the 5’ and 3’ ends of the full-length RNA strand and referred to as stem 1—could be reduced to five base pairs (bp) without compromising the binding and fluorescence of the fluorophore [42]. In order to convert the full-length aptamer into a bifurcated system we eliminated the terminal hairpin loop (which was subsequently replaced with a loop from the class I ligase ribozyme to create a binding site for the crystallization chaperone Fab BL3-6). This second closing stem (stem 2), on the opposite side of the aptamer core and which also functions to stabilize the formation of the aptamer’s binding pocket, was shortened to seven bps for initial testing (Figure 1B). Visual inspection of the model built using the Swiss PDB-Viewer [39] revealed that the nanoring could readily accommodate a 5-bp stem adjacent to the two uracil bulge near the binding pocket. Each strand of the minimized split-aptamer was tethered to one of the opposing helical struts of the nanoring via flexible single-stranded linkers. Our three-dimensional model based on the previously reported structures of the Spinach aptamer and the RNA nanoring indicated that linker strands of five to six nucleotides were needed to span the gap between the aptamer and nanoring struts (Figure 1B). Finally, realizing that the orientation of the linker strand exiting the nanoring depends on its nucleotide position within the nanoring stem, modeling revealed that grafting the linker strands on the sixth nucleotide from 5’ end of each of two struts of the nanoring directed the formation of the aptamer toward the interior of ring.

Using the visually-constructed three-dimensional model as our guide, we tested a small set of nanoring/split-aptamer systems with variable stem and linker lengths. We evaluated the fluorescence of the ring strands containing the split-aptamer sequences in the presence and absence of the peripheral helical struts responsible for complete nanoring formation. Our results showed that the nanoring/aptamer system possessing stem lengths of five base pairs on one side of the aptamer (stem 1) and six base pairs on the other side (stem 2)—in conjunction with linkers of six nucleotides (referred to as 5bp/6bp/6nt)—produced the greatest difference between ring and split-aptamer only assemblies (Figure 1D). In both cases where a longer stem was used, the fluorescence associated with the ring was lower. This data suggests that the longer stem lengths may have been sterically hindered within the interior of the ring. This is partially corroborated by the fact that 5bp/7bp/5nt exhibited higher fluorescence as a dimer than 5bp/6bp/6nt as expected given the longer stem but was lower when placed in the context of the ring.

### 3.2. Optimization and Assessment of Split-Spinach Variants

**Figure 2:**
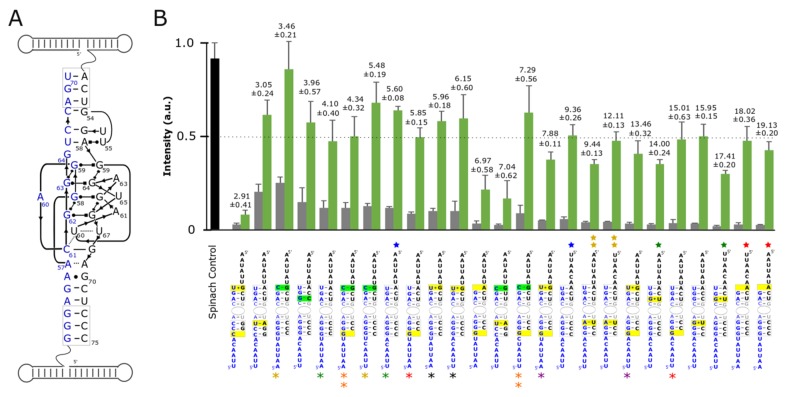
Optimization of split-Spinach as reporter for RNA nanoring assembly. (**A**) 2D diagram of split-Spinach aptamer core (adapted from Ouellet Front. Chem. 2016) [16] (**B**) Fluorescent data of variant split-Spinach sequences tested normalized to unimolecular Spinach control (black bar). Green bars indicate fluorescence associated with the fully-assembled nanoring while grey bars represent fluorescence associated with the incubation of the alpha and delta strands containing the split aptamer alone. Variants are ranked according to their respective sensitivities (i.e.. ratio of fluorescence in ring/fluorescence of dimer shown above the green and grey bars). Split-Spinach variants that differ from each other in the linker sequences are identified by colored asterisks (alpha strand) or stars (delta strand). Pairs that differ by only a single linker are identified by the same color. The number of asterisks or stars indicate the identity of the particular linker strand used. Data is based on a minimum of three trials. The error bars represent the standard deviation associated with each collection of measurements.

In the ideal case, the functional nanoring/split-aptamer would possess the ability to bind DFHBI and fluoresce only after all six strands of the nanoring were present and able to assemble into the complete nanoring. We theorized that if we wanted to rely on the split-aptamer to identify the assembly of contiguous RNA strands not directly connected to the aptamer core then we had to destabilize the split-aptamer’s propensity to assemble and create a functional aptamer on its own. Building off of our initial results, we set out to improve upon the 5bp/6bp/6nt version of the split-aptamer to provide maximum sensitivity for fully-assembled nanorings over partially-assembled ones. We hypothesized that just the right degree of destabilization in the aptamer stems could allow the formation of the nanoring to play a greater factor in promoting the formation of a functional split-aptamer which would in turn function to minimize the fluorescent signal induced by incomplete or partial assemblies. With this goal in mind, we created variants of the split-Spinach aptamer with different base pairs in the two stems surrounding the binding pocket—seeking to find a split-aptamer system that abided by a Goldilocks-like principle: just stable enough, but not too stable. We targeted the three base pairs formed at the 5’/3’ interfaces of both respective aptamer halves as prime locations to alter stem stabilities without compromising the aptamer core (Figure 2A). We theorized that they were far away from the aptamer core that their alteration could affect aptamer core stability while having a minimal effect on or interference with DFHBI binding. Base pairs were intentionally mutated and/or deleted at these positions with the goal of destabilizing the aptamer in order to prevent its functional formation in the absence of the supporting ring struts. In each case, the various split-aptamer variants were evaluated by their fluorescence intensities—normalized to the fluorescent intensity of the full-length Spinach aptamer as a control.

As a means of judging aptamer sensitivities, assemblies involving all ring strands were compared to those consisting of just the two ring strands possessing each half of the split-aptamer (alpha and delta strands) in the absence of the nanoring’s remaining supporting struts (beta, gamma, epsilon, and zeta strands respectively) (Figure 2). In order to compare and assess the sensitivity of the different variant combinations, we calculated the ratio of fluorescence of the rings to their corresponding dimers without the supporting struts. We hypothesized that substituting stronger interacting base pairs with weaker ones (i.e.. replacing GC bps with AU or GU bps) or disrupting base pairs by removing nucleotides in these two regions would destabilize the aptamer and thereby provide greater split-aptamer sensitivity over stems with increased stabilities. Generally, this hypothesis held true. In all cases where the overall stability of the split-aptamer was increased by replacing an AU bp with a GC bp the resulting variants showed higher fluorescent signals in the context of fully-assembled nanoparticles—with one combination showing nearly the same intensity as the single full-length Spinach control. The sensitivity of these stabilized systems however was generally lower than their destabilized counterparts (Figure 2B). This was due to the fact that the fluorescent signals from the dimers also increased (and in greater proportion than that of the fully formed ring systems). In most cases where the stems were destabilized, the split-aptamer showed markedly lower signals for alpha and delta strands in the absence of the four ring struts as desired. In the most extreme instances (e.g., when a nucleotide was removed from the 3’ end of each strand to remove a base pair on each side of the aptamer) the fluorescence signal associated with both the assembled ring and dimer were significantly diminished. In other situations however, the dimer signal decreased without affecting the signal from the fully-assembled ring (e.g., in cases where a GC in stem 1 was removed or replaced with an AU or GU). It is also worth noting that attempts to invert all three base pairs in stem 1 produced lower sensitivities and/or lower overall fluorescence signal in all but one case. In a few instances, attempts to destabilize stem 2 actually increased the split-aptamer’s fluorescence intensity associated with both the dimers and the assembled rings. For example, the introduction of a G at position 51 of the delta strand (changing an AU bp to a GU bp) showed increased fluorescence over the initial 5bp/6bp/6nt model (Figure 2B). This instance suggests that stem stability alone is not the only factor responsible for the split-aptamer’s performance within the context of the nanoring. Other aspects such as folding dynamics and the secondary structure of each strand is also thought to be important.

In addition to varying the composition of base pairs around the aptamer’s core we also explored the way in which different linker sequences could affect split-Spinach function. The linker sequences make up an important design element because they represent the only truly unconstrained sequence space associated with each split-aptamer strand (of course, in terms of the whole system the nucleotide sequences associated with the helical struts could also be altered by covariation of base pairs they are thought to have little to no influence on the aptamer system as a whole). We postulated that the primary influence linker sequences could have on the reporter system’s performance was through its effect on the individual strand’s secondary structure. We reasoned that linkers which were able to fold in a way that partially sequestered the portions of the split-aptamer involved in forming the stabilizing helixes could provide further sensitivity with regard to distinguishing between the presence and absence of fully-assembled nanorings. For example, in the absence of the ring the partially blocked strands would have a more difficult time forming a functional aptamer than when they are brought in close proximity due to the assembly of the nanoring. In terms of their overall design, we intentionally relied predominately on uracils (for flexibility) and adenines (to avoid unintended pairings with the multiple guanine’s associated with the aptamer core). We evaluated the folding of candidate sequences in silico using Mfold in order to find the potential secondary structures associated with each unique sequence (see Supporting Information) [41]. Generally, we found that additional secondary structure resulted in increased sensitivity. For example the addition of a GC bp to stem 2 produced the highest fluorescent signal—on par with the full the length aptamer but it lacked sensitivity because the fluorescent signal of the dimer was also the highest out of any of the constructs tested. By introducing a linker sequence that predicted a more robust secondary structure, the overall sensitivity increased by about 60% (from 3.46 to 5.48) (See supporting information, Figure S2). In other cases, strands with similar secondary structures exhibited different sensitivities—suggesting that other factors like individual folding pathways also provide subtle effects. It is clear however that the stability of the stems surrounding the aptamer core had the largest influence on a combination’s overall performance.

**Figure 3:**
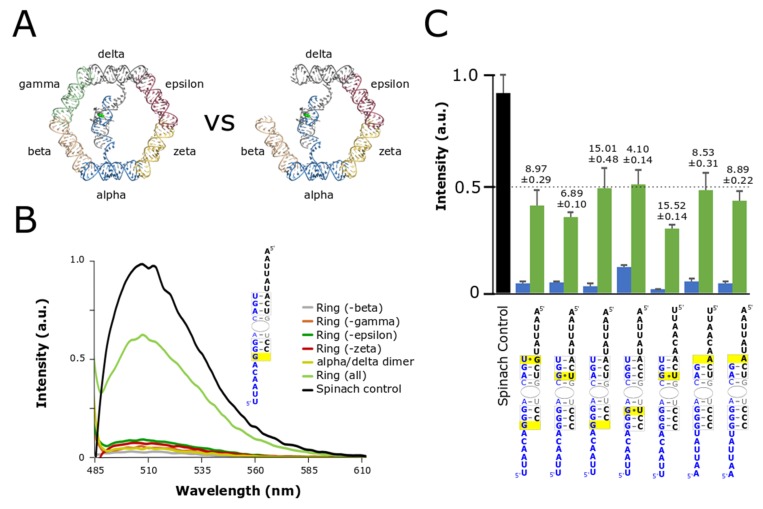
Comparison of fully- and partially-assembled nanorings. (**A**) In order to function as an effective tool for verifying and monitoring nanoring assembly the system needed to be able distinguish between whole and incomplete or partially-assembled nanorings. (**B**) Fluorescent data comparing fully-assembled to nanorings missing one of the four supporting struts (beta, gamma, epsilon, or zeta). (**C**) Summary of average fluorescent values for whole nanoring assemblies and partial assemblies compared to Spinach control where the green bar represents the complete nanoring and the blue bar represents the partially-assembled nanoring. The ratio of fluorescence of ring to partially-assembled (ring missing one strut) is shown above each plot. Data is based on a minimum of three trials. The error bars represent the standard deviation associated with each collection of measurements.

After evaluating stem stabilities and linker contributions we identified several sequence combinations that provided at least a 10-fold increase in signal between the split-aptamer in the presence of all the ring components compared to split-aptamer strands incubated by themselves with the highest ratio reaching nearly a 20-fold fluorescence gain. Following these results we evaluated the ability for the split-aptamer system to distinguish between fully- and partially-assembled nanorings. Partially-assembled rings (composed of five out of the six nanoring struts) were expected to produce a higher fluorescent signal than the split-aptamer dimers alone because, like the fully-assembled nanorings, they provide a physical conduit for the split-aptamer strands to be brought together. Previous evaluation of the nanoring however showed that partially-assembled rings are not as thermodynamically stable as fully-assembled rings and so we postulated that the split-aptamer system should be able to show some level of discrimination between the two [37] In order to evaluate the utility of split-aptamer/nanoring system further, we selected the seven most sensitive combinations (by comparing fully-assembled and split-aptamer dimers only) for further assessment in the context of partially-formed nanorings (Figure 3).

Evaluation of the partially-assembled rings revealed a number of interesting insights (Figure 3). In the first case, the strength of the fluorescence signal of the partially-assembled rings was found to be influenced by the precise identity of the missing strut. Absence of the same strut did not universally increase fluorescence across different combinations. We found that each particular split-aptamer combination had its own characteristic profile with regards to the missing strut (Figure S3). For example, for some combinations the absence of the epsilon strut produced routinely produced the highest signal while for others the highest signal involved the absence of the gamma, or zeta strut. Given the variances observed between the different split-aptamer systems we chose to compare the fluorescent response associated with the highest partially-assembled nanoring signal within each variant system—providing a worst-case scenario in terms of background signal against the fully-assembled nanoring. Secondly, while the partially-assembled constructs generally produced higher fluorescence signals than the dimer alone, we identified two split-aptamer variants that showed virtually no overall increase in fluorescent signal for partially-assembled rings compared to the dimer. In these two cases, the sensitivity of fully- over partially-assembled remained approximately 15-fold more sensitive for the fully-assembled nanoring over the partially-assembled one (Figure 3C).

### 3.3. Co-Transcriptional Assembly

A distinct advantage of RNA-based nanoparticles relates to their ability to self-assemble isothermally directly from their transcription via DNA templates [6,18,43]. This ability, paired with the rise of new RNA aptamer/fluorophore pairs has opened the door for a variety of in vitro applications involving the visualization of RNA transcripts [14,15,17,18,23,44]. The RNA nanoring, in particular, offers an attractive scaffolding platform for further functionalization and the development of high-throughput, automated medicine [6,45]. In order to evaluate the robustness of the split-aptamer/nanoring system we looked at its sensitivity with respect to fully- and partially-assembled nanorings directly resulting from the transcription of the individual DNA templates. Initial experiments evaluated the split-aptamer’s performance from equimolar mixtures of its composite RNA strands. In these cases RNA assembly was carefully controlled via a snap-cooling protocol to ensure proper folding of individual components (see Material and Methods). In the case of co-transcriptional assembly, equimolar concentrations of DNA templates were added to a transcription mixture along with T7 RNA polymerase. The resulting transcripts were left to self-assemble in the mixture during the course of the experiment.

**Figure 4:**
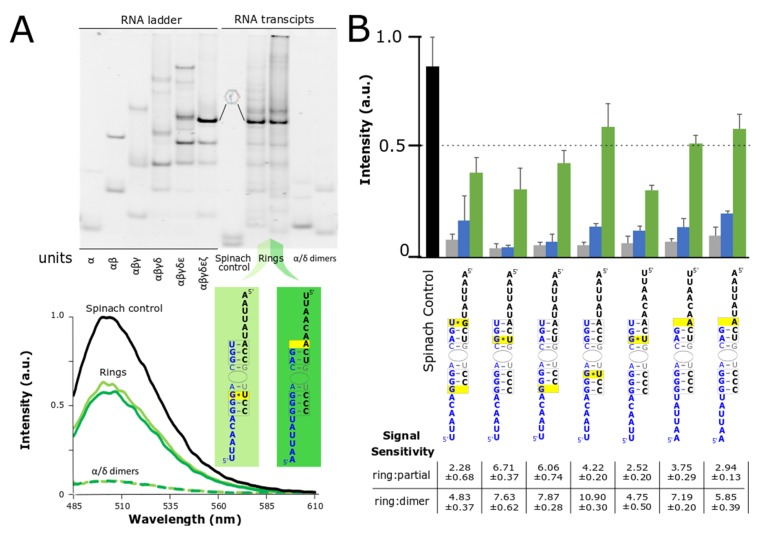
Co-ranscriptional assembly of nanorings monitored by native PAGE and fluorescent spectroscopy. (**A**) Native PAGE gel (1× HEPES and 2 mM Mg^2+^) was used to reveal the composition of nanoring assemblies formed in transcription mix as compared to an RNA ladder that was snapped cooled (top). Fluorescent values associated with the nanoring products were normalized to the Spinach control also formed via transcription (bottom). (**B**) Average fluorescent data for top split-Spinach variants assembled during transcription with ratio of fluorescent signal of fully-assembled ring (green bar) to partially-assembled ring (blue bar) and fully-assembled ring to dimer (grey bar). Data is based on a minimum of three trials. The error bars represent the standard deviation associated with each collection of measurements.

We evaluated the self-assembly of the split-aptamer modified RNAs by fluorescence spectroscopy and by gel electrophoresis (Figure 4). Native PAGE gels (40 mM HEPES, pH 8.2 buffer and 2 mM Mg(OAc)_2_) were used to compare the resulting RNA assemblies of the transcription mixture with an RNA ladder prepared via snap-cooled assembly of the corresponding nanoring. Native PAGE reveals that the fully-assembled nanorings constitute the primary assembly product (Figure 4A). The fluorescent signal associated with dimers, partially-assembled, and fully-assembled nanorings supports this same outcome. While the signaling ratio between fully-assembled and partially-assembled rings was on the whole lower, the fully-assembled rings achieved between 2- and nearly 7-fold increase in fluorescence over the partially-assembled rings. Fully-assembled nanorings have been shown to be more chemically and thermodynamically stable than partially-assembled ones [37]. Given that the partially-assembled nanorings would have much shorter half-lives and be less prone to form, we postulate that sensitivity of the fully-assembled nanoring over the partially-assembled ones could be increased in certain environments. More impressively (considering fluorescence is triggered by the folding and assembly of six independent strands versus a single transcript) the fluorescent signal of three of the ring systems produced signal levels that remained at over 50% of the single-transcript Spinach aptamer. This is particularly remarkable given the fact that maximum fluorescence is achieved only by the production and assembly of six individual strands compared to the transcription and intramolecular folding of the single control strand. Because the fluorescent output of the split-aptamer remains quite strong compared to the full-length Spinach control, it shows promise as a reporter for nanoparticle formation from isothermal assembly of transcription products. Collectively, we believe that these results demonstrate the system’s potential for use in high-throughput assembly and other applications.

## 4. Conclusions

The design and characterization of an aptamer/fluorophore integrated RNA nanoparticle system demonstrates the ability to identify perihperal tertiary interactions between assembled RNA strands of the six-membered RNA nanoring. While many of the particular design constraints discussed above pertain to the specific system at hand, our work reveals a few general principles relating to the design of future split-aptamer systems. During the course of our design we observed that stem stability and performance of any particular sequence (characterized by the various linker designs) require careful testing. This suggests that full-scale automation of the design process remains elusive and that the design of new sytems will undoubtedly contain their own particular parameters which will also require their own experimental optimization. Simply put, the overall performance of variants is and remains context dependent and very difficult to predict. In this regard, even though in vitro studies provide a baseline for assessing the behavior of the a self-assembling nanoparticle/split-aptamer/fluorophore system, evaluation in increasingly complex environments remain necessary for validation and further refinenment. Modeling and design provide an essential starting point but due to myriad of parameters, experimental refinement remains absolutely necessity. Our work also shows that the destablization of stems surrounding the aptamer core generally provide increased sensitivity between fully- and partially assembled nanoparticles. The same is likely to be true for future developments with different nanoparticles—where the goal or purpose of nanoparticle assembly functions, at least in part, to restore and/or compensate the intentionally diminished stabilities of the altered stems.

The split-aptamer/nanoparticle system demonstrates the ability, not only to monitor the assembly of multiple strands, but the ability to monitor the assembly of RNA strands not directly linked to the aptamer units. To our knowledge this is the first system developed that demonstrates the ability to detect the formation of tertiary interactions on contiguous strands of RNA. We demonstrate further that fluorescent signaling associated with fully-assembled nanorings can be maximized over the partially assembled split-aptamer system associated with two or five strand mixtures. Given its ability to assemble isothermally from DNA templates, we propose that the co-transcriptional assembly of the split-aptamer/nanoring system provides a robust platform for the further development of a variety of automated and/or high-throughput applications. The nanoring’s four other struts are readily functionalizable and because the nanoring’s helical struts can be increased a full helical turn to accommodate a larger interior space, it is possible that more complex aptamer systems may be able to be incorporated (such as the addition of secondary modular aptamer domain). Finally, fluorescent reporting of fully-formed nanorings provides instant feedback for the previously proposed automated assembly of therapeutic RNA nanoparticles [45].

## Supplementary Materials

The following are available online at https://www.mdpi.com/2079-4991/9/3/378/s1, Figure S1: 2D diagram of aptamer integrated nanoring, Table S1: List of sequences tested, Figure S2: Predicted secondary structures. Figure S3: Fluorescent profile of partially-assembled nanorings.
