# Supplementary material for: Optimization of the Split-Spinach Aptamer for Monitoring Nanoparticle Assembly Involving Multiple Contiguous RNAs
Source: Nanomaterials (Basel). 2019 Mar 6;9(3):378. doi: 10.3390/nano9030378 (PMC6474029; doi:10.3390/nano9030378)
Supplement: Supplementary file 1 [file nanomaterials-09-00378-s001.pdf]

Optimization of the Split-Spinach Aptamer for Monitoring Nanoparticle Assembly Involving Multiple Contiguous RNAs

Jack M. O'Hara [1], Dylan Marashi [1], Sean Morton [1], Luc Jaeger [2] and Wade W. Grabow [1,*]

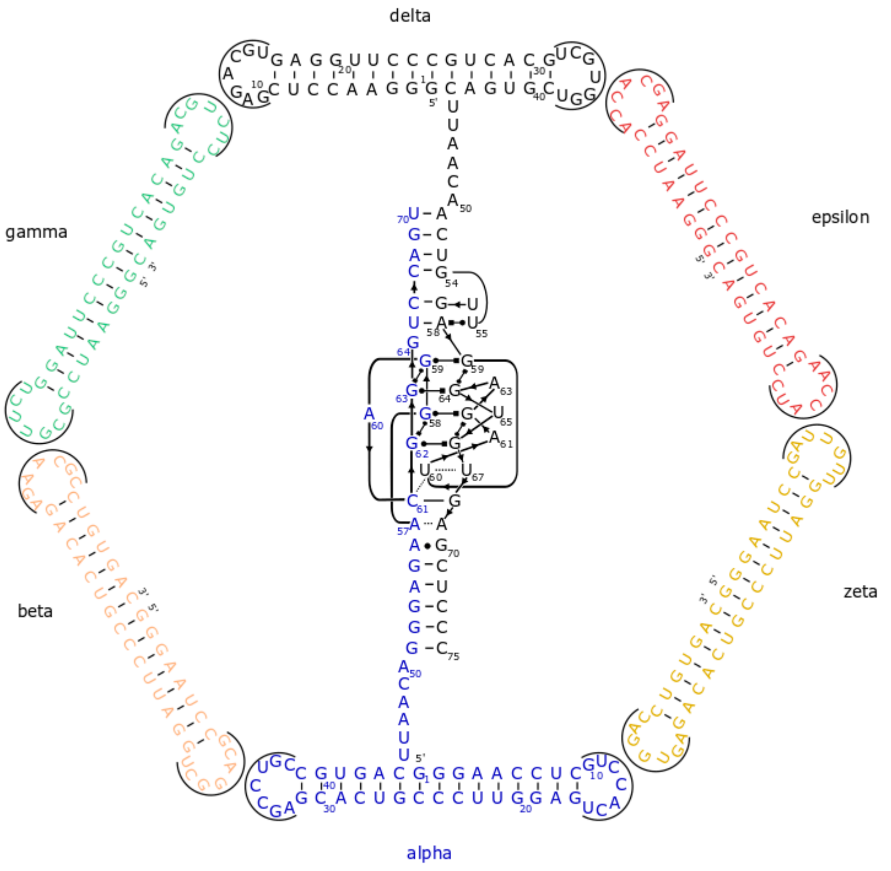

Figure S1. 2D sequences associated with the 5bp/6bp/6nt version of the split-aptamer integrated nanoring system.

Full-length Spinach control
5′-GGGAGAAGGACGGGUCCAGUGCGAAACACGCACUGUUGAGUAGAGUGUGAGCUCCC-3′

Ring Struts
beta
5'-GGGAAcCuCGCAGGCUGaGgUUCCCGUCACGAGAACGCCGUGAC-3′

gamma
5′-GGGAAcCuCGCGUUCUGaGgUUCCCGUCACGACGUCUCCGUGAC-3′

epsilon
5′-GGGAAcCuCACCACGAGaGgUUCCCGUCACGAACCAUCCGUGAC-3′

zeta

5′-GGGAAcCuCGAUGGUUGaGgUUCCCGUCACGAGUGGACCGUGAC-3′

Stem/linker exploration

5bp/6bp/6nt version of the split-aptamer

5′GGGAACCUCGUCCACUGAGGUUCCCGUCACGAGCCUGCCGUGACUUAACAGGGAGAAGGACGGGUCCAGU3′

5′GGGAACCUCGAGACGUGAGGUUCCCGUCACGUCGUGGUCGUGACUUAACAACUGUUGAGUAGAGUGUGAGCUCCC3′

5bp/7bp/6nt version of the split-aptamer

5′ GGGAACCUCGUCCACUGAGGUUCCCGUCACGAGCCUGCCGUGACUUAAcAgGGAGAAGGACGGGUCCAGU - 3′

5′ GGGAACCUCGAGACGUGAGGUUCCCGUCACGUCGUGGUCGUGACUUAAcACACUGUUGAGUAGAGUGUGAGCUCCc-3′

5bp/7bp/5nt version of the split-aptamer

5′ GGGAACCUCGUCCACUGAGGUUCCCGUCACGAGCCUGCCGUGACUUAAAgGGAGAAGGACGGGUCCAGU - 3′

5′ GGGAACCUCGAGACGUGAGGUUCCCGUCACGUCGUGGUCGUGACUUAAACACUGUUGAGUAGAGUGUGAGCUCCc-3′

alpha-strands tested

GGG-AGU

5′GGGAACCUCGUCCACUGAGGUUCCCGUCACGAGCCUGCCGUGACUUAACAGGGAGAAGGACGGGUCCAGU3′

GGA-AGU

5′GGGAACGUCGUCCACUGACGUUCCCGUCACGAGCCUGCCGUGACUUAACAGG AGAAGGACGGGUCCAGU3′

GGG-GGU

5′GGGAACGUCGUCCACUGACGUUCCCGACACCAGCCUGCGGUGUCUUAACAGGGAGAAGGACGGGUCCGGU3′

GGG-AGU.1

5′GGGAACCUCGUCCACUGAGGUUCCCGUCACGAGCCUGCCGUGAC GGGAGAAGGACGGGUCCAGU3′

GGG-AG_.1

5′GGGAACCUCGUCCACUGAGGUUCCCGUCACGAGCCUGCCGUGAC GGGAGAAGGACGGGUCCAG 3′

GGG-AGC.2

5′GGGAACCUCGUCCACUGAGGUUCCCGUCACGAGCCUGCCGUGACUUAACUGGGAGAAGGACGGGUCCAG 3′

GGG-AGC.3

5′GGGAACCUCGUCCACUGAGGUUCCCGUCACGAGCCUGCCGUGACUUAAUCGGGAGAAGGACGGGUCCA
G 3′

GG-AGC.1
5′GGGAACCUCGUCCACUGAGGUUCCCGUCACGAGCCUGCCGUGAC GGGAGAAGGACGGGUCC
AG 3′

CCU-AGC
5′
GGGAACGUCGUCCACUGACGUUCCCGUCACGAGCCUGCCGUGACUUAACA AGAAGGACGGGUCCAG
C3′

CCC-GGU
5′
GGGAACGUCGUCCACUGACGUUCCCGUCACGAGCCUGCCGUGACUUAACACCCAGAAGGACGGGUCCGG
U3′

CCC-AGU
5′
GGGAACGUCGUCCACUGACGUUCCCGUCACGAGCCUGCCGUGACUUAACA AGAAGGACGGGUCCAG
U3′

CCU-AGU
5′GGGAACGUCGUCCACUGACGUUCCCGUCACGAGCCUGCCGUGACUUAACA AGAAGGACGGGUCCA
GU3′

CCU-AGU
5′
GGGAACGUCGUCCACUGACGUUCCCGUCACGAGCCUGCCGUGACUUAACA AGAAGGACGGGUCCAG
U3′

delta-strands tested
ACU-CCC5′
GGGAACCUCGAGACGUGAGGUUCCCGUCACGUCGUGGUCGUGACUUAACAACUGUUGAGUAGAGUGUG
AGCUCCC3′

ACU-UCC5′
GGGAACCUCGAGACGUGAGGUUCCCGUCACGUCGUGGUCGUGACUUAACAACUGUUGAGUAGAGUGUG
AGCUUCC3′

ACU-CCC.1
5′GGGAACCUCGAGACGUGAGGUUCCCGUCACGUCGUGGUCGUGAC ACUGUUGAGUAGAGUGU
GAGCUCCC -3′

ACU-UCC.1
5′GGGAACCUCGAGACGUGAGGUUCCCGUCACGUCGUGGUCGUGAC ACUGUUGAGUAGAGUGU
GAGCUUCC -3′

ACC-UCC.1
5′GGGAACCUCGAGACGUGAGGUUCCCGUCACGUCGUGGUCGUGAC AC GUUGAGUAGAGUGU
GAGCU CC -3′
ACC-CCC.1
5′GGGAACCUCGAGACGUGAGGUUCCCGUCACGUCGUGGUCGUGAC AC GUUGAGUAGAGUGU
GAGCUCCC -3′

GCU-CCC.1
5′GGGAACCUCGAGACGUGAGGUUCCCGUCACGUCGUGGUCGUGAC CUGUUGAGUAGAGUGU
GAGCUCCC -3′

GCU-CC_.1
5′GGGAACCUCGAGACGUGAGGUUCCCGUCACGUCGUGGUCGUGAC CUGUUGAGUAGAGUGU
GAGCUCC -3′

ACU-CC_.1
5′GGGAACCUCGAGACGUGAGGUUCCCGUCACGUCGUGGUCGUGAC ACUGUUGAGUAGAGUGU
GAGCUCC. -3′

ACU-CC_.1
5′GGGAACCUCGAGACGUGAGGUUCCCGUCACGUCGUGGUCGUGAC ACUGUUGAGUAGAGUGU
GAGCUCC -3′

ACU-GGG.2
5′GGGAACCUCGAGACGUGAGGUUCCCGUCACGUCGUGGUCGUGAC A ACUGUUGAGUAGAGUGU
GAGCUGGG -3′

ACU-AGG.2
5′GGGAACCUCGAGACGUGAGGUUCCCGUCACGUCGUGGUCGUGAC A ACUGUUGAGUAGAGUGU
GAGCUAGG -3′

GCU-GGG.2
5′GGGAACCUCGAGACGUGAGGUUCCCGUCACGUCGUGGUCGUGAC A GCUGUUGAGUAGAGUGU
GAGCUGGG -3′

GUU-GGG.2
5′GGGAACCUCGAGACGUGAGGUUCCCUGCACGUCGUGGUCGUGCA A GUUGUUGAGUAGAGUGU
GAGCUGGG -3′

GUU-AGG.2
5′GGGAACCUCGAGACGUGAGGUUCCCUGCACGUCGUGGUCGUGCA A GUUGUUGAGUAGAGUGU
GAGCUAGG -3′

GCU-GG_.2
5′GGGAACCUCGAGACGUGAGGUUCCCGUCACGUCGUGGUCGUGAC A GCUGUUGAGUAGAGUGU
GAGCUGG. -3′

GCU-AGG.3
5′GGGAACCUCGAGACGUGAGGUUCCCGUCACGUCGUGGUCGUGAC A GCUGUUGAGUAGAGUGU
GAGCUAGG -3′

Table S1. Complete list of sequences used in study. Green highlights represent the portion of the Spinach aptamer that was appended to the alpha strand of the nanoring. Yellow highlights represent the portion of the aptamer appended to the delta strand. Light blue highlights show the kissing loop sequences associated with the programmable nanoring.

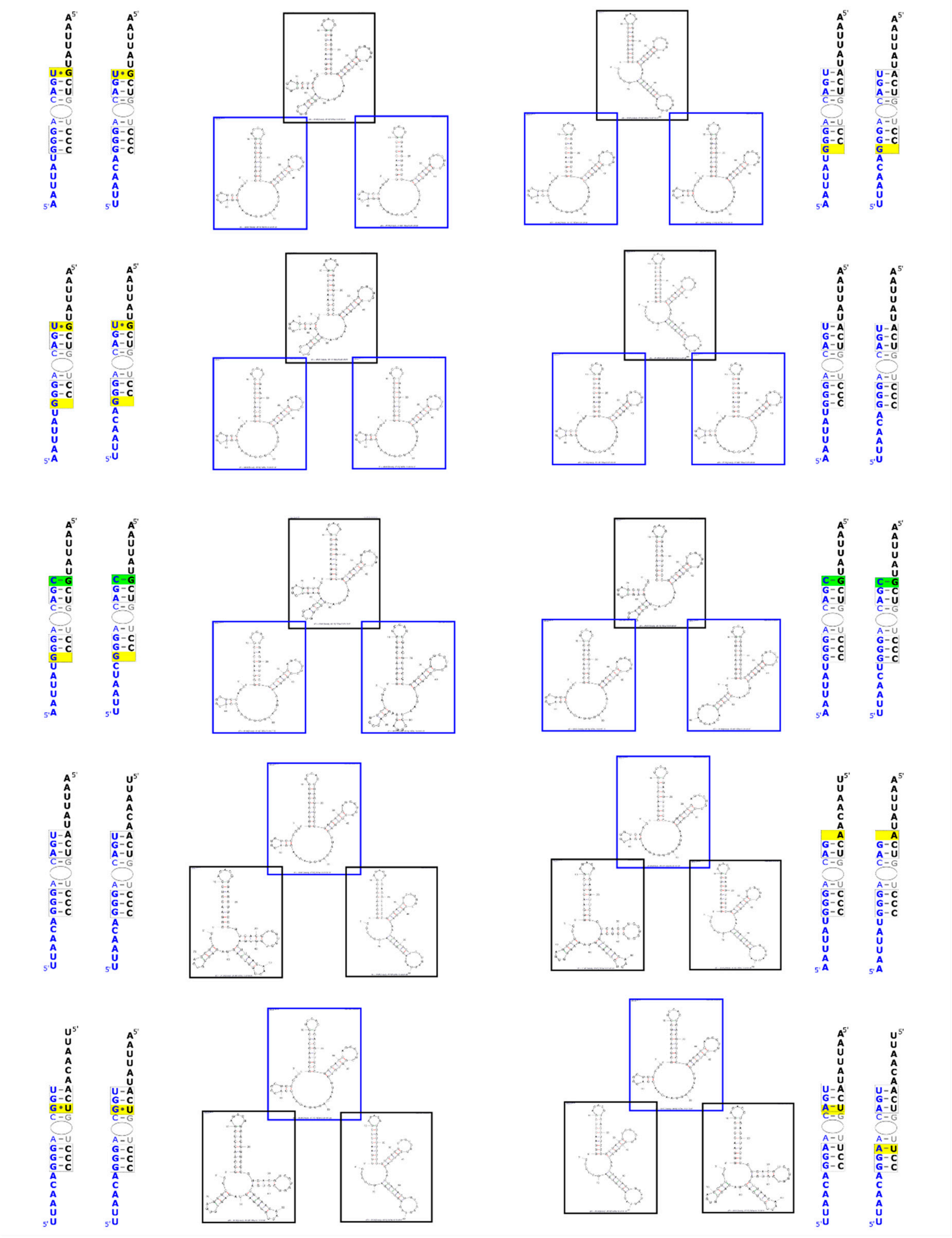

Figure S2. Predicted secondary structures for individual strands based on linker variants. The 2D structures were generated using the mfold server.1 The two hairpins of 9- and 6-bp represent the struts of the nanoring. The remaining portion represent the most stable predicted secondary structure of the linker and split-aptamer region. The predicted structures were evaluated to determine whether the linker prevented or facilitated additional secondary structure to sequester the aptamer strand.
